# Porengeführte Injektion: Eine einfache schmerzarme Technik bei der Lokalanästhesie

**DOI:** 10.1111/ddg.16006_g

**Published:** 2026-06-04

**Authors:** Felipe Bochnia Cerci, Isadora R. Scaburi, Umer Nadir, Stanislav N. Tolkachjov

**Affiliations:** ^1^ Clínica Cepelle Curitiba Brazil; ^2^ Dermatology Service Hospital Universitário Evangélico Mackenzie Curitiba Brazil; ^3^ Epiphany Dermatology, Dallas, Texas; ^4^ Texas A&M College of Medicine, Dallas, Texas; ^5^ Department of Dermatology The University of Texas at Southwestern Medical Center, Dallas, Texas; ^6^ Division of Dermatology Baylor Scott & White, Dallas, Texas

**Keywords:** Dermatologische Chirurgie, Hautkrebs, Lokalanästhetikum, Mikrographische Mohs‐Chirurgie, Poren‐gesteuerte Injektion, Dermatologic surgery, local anesthetic, Mohs micrographic surgery, pore‐guided injection, skin cancer

## EINLEITUNG

Lokalanästhesie wird bei vielen dermatologischen Eingriffen eingesetzt, darunter Biopsien, Kauterisation, Exzision und chirurgische Wundrekonstruktion.^1^ Die Injektion des Lokalanästhetikums wird oft als der schmerzhafteste Teil dieser Eingriffe angesehen und ist häufig das Ereignis, an das sich Patienten nach dem Eingriff am meisten erinnern.^2^, ^3^ Daher ist die Verringerung der mit der lokalen Infiltrationsanästhesie während dermatologischer Eingriffe verbundenen Schmerzen sehr wichtig. Schmerzreduktion trägt direkt zur Patientenzufriedenheit bei, verringert perioperative Angst und kann die Kooperation während des Eingriffs verbessern, während gleichzeitig das Risiko psychologischer Traumata und zukünftiger Abneigungen gegen medizinische Behandlungen verringert wird.^4^

Das erste Einführen der Nadel bei der Lokalanästhesie kann schmerzhaft sein, insbesondere in Bereichen des Gesichts mit hoher Nervendichte. Mehrere Methoden zur Reduktion der mit Lokalanästhesie verbundenen Schmerzen wurden bereits beschrieben. Wir demonstrieren hier eine Technik zum Einführen der Nadel durch eine Pore, die unserer Meinung nach eine zusätzliche Methode sein kann, um den Patienten weniger Schmerzen zu bereiten.

## TECHNIK

**ABBILDUNG 1:**
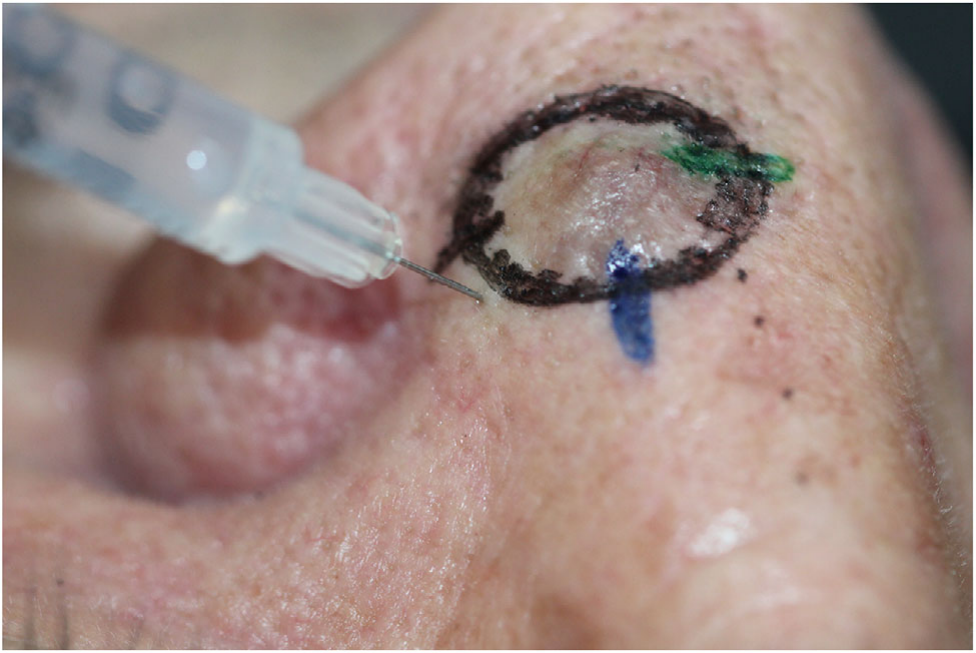
Klinische Demonstration der Nadeleinführung durch eine Nasenporenöffnung.

Nach routinemäßiger Antisepsis und Identifizierung einer geeigneten Pore für die Injektion wird die Nadel in einem Winkel von 90 Grad durch die Pore eingeführt (Abbildung 1). Für eine bessere Sichtbarkeit vor und während der Injektion können Dermatoskopie, Lesebrillen (*Cheaters*) oder Lupenbrillen verwendet werden.

## DISKUSSION

**ABBILDUNG 2:**
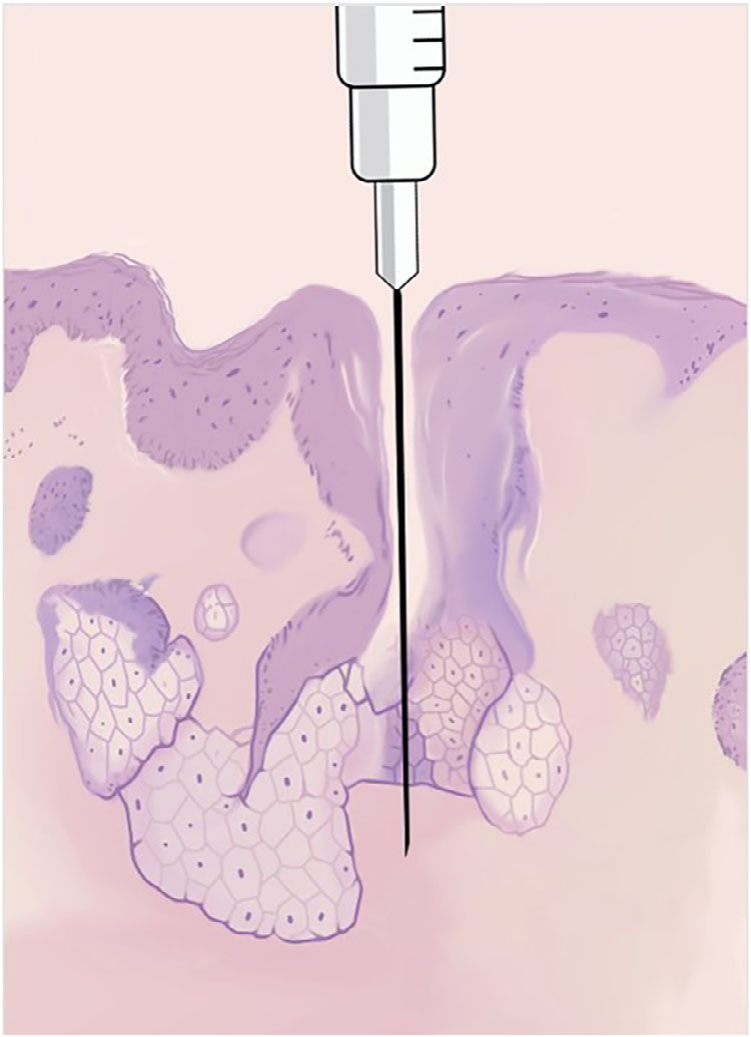
Schematische histologische Darstellung der „Abkürzung” beim Einführen der Nadel durch die Pore.

Die Autoren halten diese Technik für nützlich in seborrhoischen Arealen des Gesichts, insbesondere am distalen Drittel der Nase. Das Einführen der Nadel für die Lokalanästhesie durch eine Pore kann zu weniger Schmerzen führen, da es eine bereits vorhandene Öffnung in der Haut nutzt und dadurch das umliegende Gewebe weniger stark beeinträchtigt. Dieser Ansatz reduziert mechanische Verletzungen des dichten Kollagennetzwerks und der Nervenenden in der Dermis, die in erster Linie Schmerzsignale übertragen (Abbildung 2). Durch die Begrenzung des Kontakts mit Nozizeptoren in den oberflächlichen Hautschichten minimiert das Verfahren den anfänglichen nozizeptiven Reiz. Darüber hinaus kann die Verwendung der Pore als natürlicher Zugang lokale Entzündungen und Gewebetraumata verringern und so zu einer angenehmeren Erfahrung für den Patienten während der Anästhesie beitragen. Andere Techniken zur Verringerung von Beschwerden, wie langsame Infiltrationsgeschwindigkeit, Verwendung einer Nadel mit kleinem Durchmesser, Verwendung einer kleinen Spritze, Gespräche mit dem Patienten und Ablenkungstechniken, sollten nicht vergessen werden.^5^, ^6^ Zukünftige Forschungen sollten die Wirksamkeit dieser Technik untersuchen und weitere Methoden zur Schmerzlinderung bei Lokalanästhesie skizzieren.

## INTERESSENKONFLIKT

S.N.T. ist Referent und Forscher für CASTLE Biosciences, Kerecis und Boehringer Ingelheim. Die anderen Autoren erklären, dass keine Interessenkonflikte bestehen.
